# A review of Duhuo Jisheng decoction mechanisms in intervertebral disc degeneration in vitro and animal studies

**DOI:** 10.1186/s13018-023-03869-4

**Published:** 2023-06-16

**Authors:** Daqian Zhou, Chao Song, Yongliang Mei, Kang Cheng, Fei Liu, Weiye Cai, Silong Gao, Zhenlong Wang, Zongchao Liu

**Affiliations:** 1 Department of Orthopedics, The Affiliated Hospital of Traditional Chinese Medicine of Southwest Medical University, Luzhou, 646000 Sichuan Province China; 2 Luzhou Longmatan District People’s Hospital, Luzhou, Sichuan Province China

**Keywords:** Duhuo Jisheng decoction, Intervertebral disc degeneration, Molecular mechanism, Traditional Chinese medicine

## Abstract

Intervertebral disc degeneration (IVDD) has become a serious public health problem, placing a heavy burden on society and the healthcare system. Its pathogenesis is not completely clear and may be closely related to mechanical damage, inflammatory factors, oxidative stress and death of nucleus pulposus cells (NPCs). The treatment of IVDD mainly includes conservative treatment and surgery. Conservative treatment is based on hormonal and anti-inflammatory drugs and massage techniques, which can relieve the pain symptoms to a certain extent, but cannot solve the problem from the root cause. Surgical treatment is mainly by removing the herniated nucleus pulposus, but it is more traumatic for IVDD patients, expensive and not suitable for all patients. Therefore, it is extremely important to clarify the pathogenesis of IVDD, to find an effective and convenient treatment and to further elaborate its mechanism of action. The effectiveness of traditional Chinese medicine in the treatment of IVDD has been well demonstrated in clinical medical research. We have been working on the Chinese herbal formula Duhuo Jisheng Decoction, which is a common formula for the treatment of degenerative disc disease. Not only does it have significant clinical effects, but it also has few adverse effects. At present, we found that its mechanism of action mainly involves regulation of inflammatory factors, reduction of apoptosis and pyroptosis of NPCs, inhibition of extracellular matrix degradation, improvement of intestinal flora, etc. However, a few relevant articles have yet comprehensively and systematically summarized the mechanisms by which they exert their effect. Therefore, this paper will comprehensively and systematically explain on it. This is of great clinical significance and social value for elucidating the pathogenesis of IVDD and improving the symptoms of patients, and will provide a theoretical basis and scientific basis for the treatment of IVDD with traditional Chinese medicine.

## Introduction

**Fig. 1:**
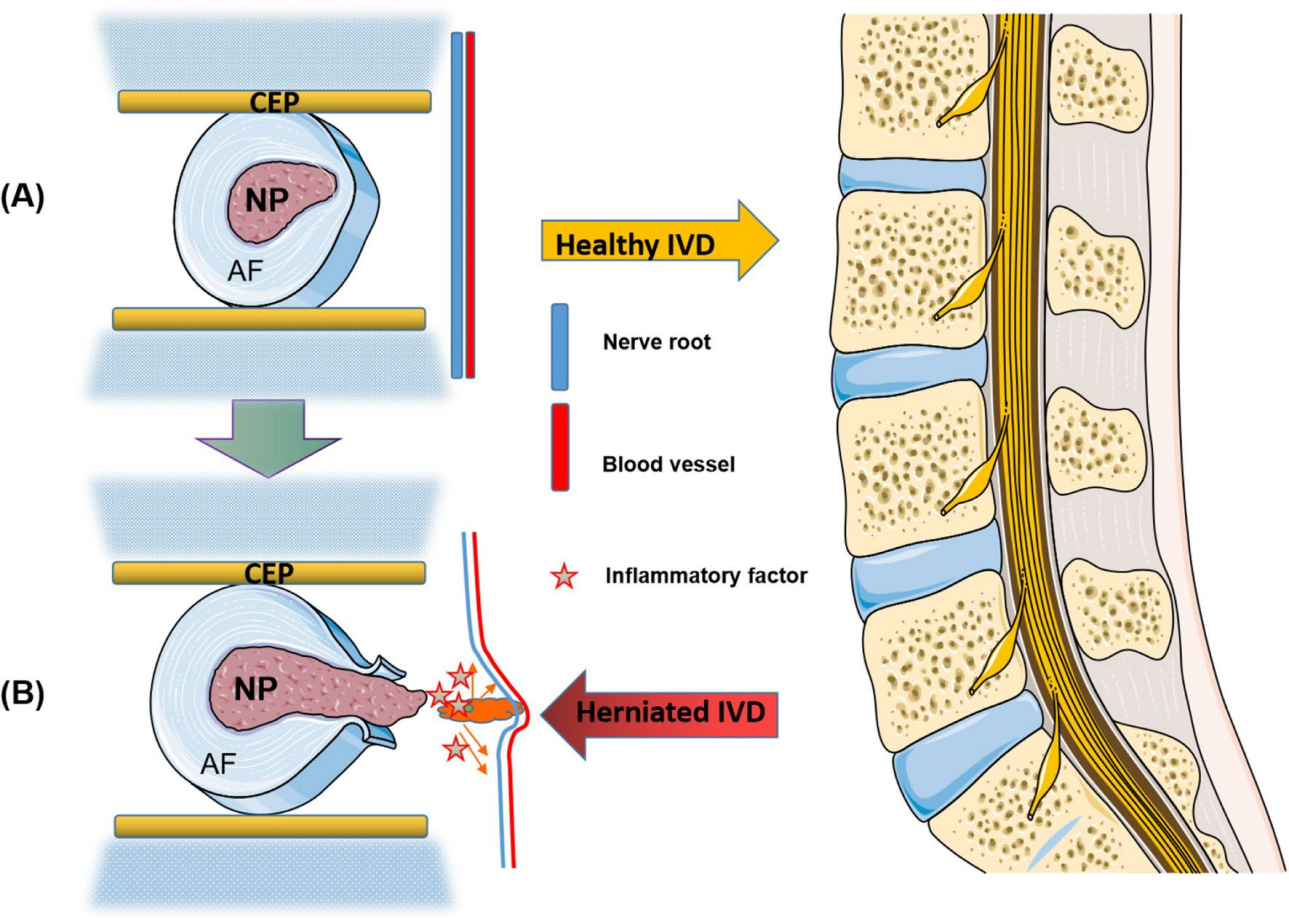
Schematic diagram of the structure of normal and degenerated discs. **A** The normal intervertebral disc is composed of the fibrous annulus (AF), cartilage end plate (CEP) and nucleus pulposus (NP), all three of which provide the basis for disc homeostasis. **B** In degenerated discs, the nucleus pulposus (NP) tissue protrudes outward to compress nerve roots and blood vessels, and inflammatory factors are released and accumulate, exacerbating the degree of disc degeneration

Intervertebral disc degeneration (IVDD) is one of the following common chronic diseases in orthopedics with low back pain as the main symptom [1], affects approximately 40% of the world's population and places a heavy burden on society and health care systems [2, 3]. As the largest lack of vascular tissue in the human body, the intervertebral disc is mainly composed of three parts: the central nucleus pulposus tissue, the fibrous rings on both sides and the upper and lower cartilage endplates. The nutrients and metabolites enter and exit mainly through semi-permeable membrane-like micropores in the cartilage plate [4]. IVDD mainly includes lumbar disc herniation (LDH) and lumbar disc degeneration (LDD). Its etiology and pathogenesis have not been fully elucidated, and the cause may be related to various factors, such as smoking, age, obesity, mechanical, genetic and nutritional factors [5]. The pathogenesis may be related to mechanical injury leading to partial or complete rupture of the intervertebral disc's annulus fibrosus, protrusion of the nucleus pulposus(NP) outward to compress nerve roots and cause a variety of symptoms, or various triggers leading to degeneration of the disc structure and formation of inflammatory irritation, oxidative stress and apoptosis and pyroptosis of the NPCs, etc. [6–8]. Currently, the treatment of IVDD patients is divided into two categories: conservative treatment and surgical treatment [9]. Early stages of the disease can be treated conservatively with drugs and physical therapy to relieve pain to some extent [10], but it fails to solve the problem from the etiology. Surgical excision of prominent NP can effectively relieve the clinical symptoms of IVDD in late stages, but not all patients who undergo surgical treatment achieve good results [11]. Therefore, it becomes extremely important to clarify the pathogenesis of IVDD so as to find a more effective treatment modality (Fig. 1).

It is well known that Chinese herbal medicine plays an important role in health maintenance in Asian countries such as China. In addition, herbal medicine has been used as a complementary and alternative treatment for patients with IVDD [12]. Many modern studies have partially demonstrated the effectiveness of herbal medicine in the treatment of IVDD, and the main mechanisms involve reduction of oxidative stress, inhibition of inflammatory response and reduction of NPCs death, etc. [6, 13]. For example, Liuwei Dihuang Decoction can inhibit the apoptosis of intervertebral disc cells by regulating Caspase-3, IL-1β and other related targets to treat IVDD. Yi-Qi-Huo-Xue-Tang delays intervertebral disc degeneration by activating autophagy and a single herbal medicine, Salvia miltiorrhiza, attenuates intervertebral disc degeneration in SD rats via antioxidant [14, 15].

Duhuo Jisheng Decoction (DHJSD) is wildly used to treat IVDD, which was firstly recorded in < Thousand-Golden-Prescriptions > , a famous TCM recipe book in the tang dynasty [16, 17]. We found that DHJSD has good clinical efficacy and can significantly reduce VAS and JOA scores in patients with lumbar disc herniation. Nowadays, clinical medical research has fully proved the effectiveness of traditional Chinese medicine in the treatment of IVDD, especially the traditional Chinese medicine compound DHJSD has been widely used in the treatment of IVDD, and its main mechanism involves reducing oxidative stress, inhibiting inflammatory response and reducing NP cell death, improving intestinal flora, etc. [18]. Although we have studied the mechanism of action of DHJSD in the treatment of IVDD in various aspects, there is no relevant article to summarize the mechanism of its effect in a comprehensive and systematic way. Therefore, we will summarize the mechanism of drug action of DHJSD in the treatment of IVDD from the following aspects.

## DHJSD regulates inflammatory factors and signaling pathways for the treatment of IVDD

Low back pain is a common clinical symptom in patients with IVDD, and anti-inflammatory and analgesic therapy is particularly important for treatment because of inflammatory factors releasing and inflammation often occurring in the process of IVDD [19]. Account to normal person, the level of inflammatory factors is higher in damaged intervertebral disc or degenerated intervertebral disc tissue, such as tumor necrosis factor alpha (TNF-α), interferon gamma (IFN-γ), nitric oxide (NO), and IL-1, IL-4, IL-6, and IL-8 which promote immune response, and induce nucleus pulposus cell degeneration and even death by participating in inflammation and proceeding immune reaction [19–21]. IL-1β and TNF-α, particularly, are positively correlated with the degree of disc degeneration [22]. These inflammatory factors primarily activate the nuclear factor-κB (NF-κB) and mitogen-activated protein kinase (MAPK) signaling pathways, which in turn cause the production of cytokines, chemokines, and extracellular matrix degradation factors in IVDD and ultimately the inflammatory waterfall cascade response [16, 18].

A large number of studies have suggest that DHJSD may inhibit the production, activity, signal transduction and receptor activity of inflammatory cytokines, and result in anti-inflammatory, pain relieving, immune regulation and anti-platelet aggregation. Mechanism of action involves inhibition of inflammatory cytokine production, inhibition of inflammatory cytokine action, inhibition of inflammatory cytokine signaling, and inhibition of receptor activity of inflammatory cytokines [23–25]. In recent years, we mainly focus on the study of the inflammatory mechanism of IVDD treated with DHJSD, and we found that DHJSD can effectively inhibit the production of inflammatory factors TNF-α and IL-1β and improve the inflammation of intervertebral disc [26]. For in-depth analysis and mechanism verification, we isolated primary NPCs from degenerated discs and normal lumbar fracture patients for in vitro culture and treated the cells with stromal cell-derived factor-1 (SDF-1) and DHJSD at different concentrations, and found that DHJSD significantly antagonized SDF-1-induced pro-inflammatory cytokine production, the results demonstrate that DHJSD may have a therapeutic effect on low back pain by reducing inflammatory cytokine formation and inhibiting the production of pro-inflammatory mediators through the SDF-1/CXCR4/NF-κB pathway [6]. In the recent study, we also demonstrated that DHJSD can inhibit the release of inflammatory factors IL-1β and TNF-α through the MAPK pathway. In summary, DHJSD can inhibit the production of inflammation-related factors such as TNF-α and IL-1, and its main mechanism may be through the regulation of inflammatory response pathways such as MAPK and NF-κB to inhibit the inflammatory response and improve IVDD.

## DHJSD regulates NPCs death in the treatment of IVDD

The intervertebral disc is mainly composed of the NP, annulus fibrous (AF) and cartilage end plate (CEP): the central nucleus pulposus is wrapped by the fibrous annulus on both sides and the upper and lower cartilage end plates [27]. NP as the internal of the IVDD, distributes the external force evenly to AF and avoids local damage caused by overload [28], The reduction of NPCs is one of the important features of IVDD [29]. The damage and reduction of NPCs in IVDD is caused by multiple factors, among which multiple cell death modalities such as apoptosis, pyroptosis, and autophagy may be the main causes [30–33]. Apoptosis requires activation of caspase-3, -8, and -9, accompanied by cleavage of the nucleus, cleavage of DNA and nucleases, and preservation of plasma membrane integrity. During apoptosis, the contents of apoptotic cells are packaged into apoptotic bodies that are phagocytosed and cleared by macrophages without causing inflammation [34]. Apoptotic mechanisms can eliminate abnormal growth and susceptibility to deterioration of cells in different pathophysiological processes, and normal apoptosis is beneficial for maintaining functional and structural stability of the intervertebral disc. However, excessive apoptosis of NPCs is one of the most common pathogenic mechanisms of IVDD [35, 36], In fact, the extent of apoptosis in IVDD specimens reached 53 ~ 73% [37].

Recent studies have shown that excessive apoptosis of NPCs is largely promoted by an imbalance of the cellular redox system in the intervertebral disc microenvironment and that intervention against oxidative stress can significantly inhibit the occurrence of apoptosis in nucleus pulposus cells [38, 39]. It has been shown that activation of the MAPK pathway is a key step in oxidative stress-related apoptosis [40], and blocking the p38/MAPK signaling pathway can inhibit the apoptosis of NPCs and prevent the development or delay the progression of IVDD [41]. The MAPK signaling pathway is an important pathway of apoptosis. p38-MAPK, extracellular signal-regulated kinase (ERK) and c-jun terminal kinase (JNK) are the three major subgroups of MAPK. Further studies revealed that DHJSD can affect p38/MAPK signaling [42].

**Fig. 2:**
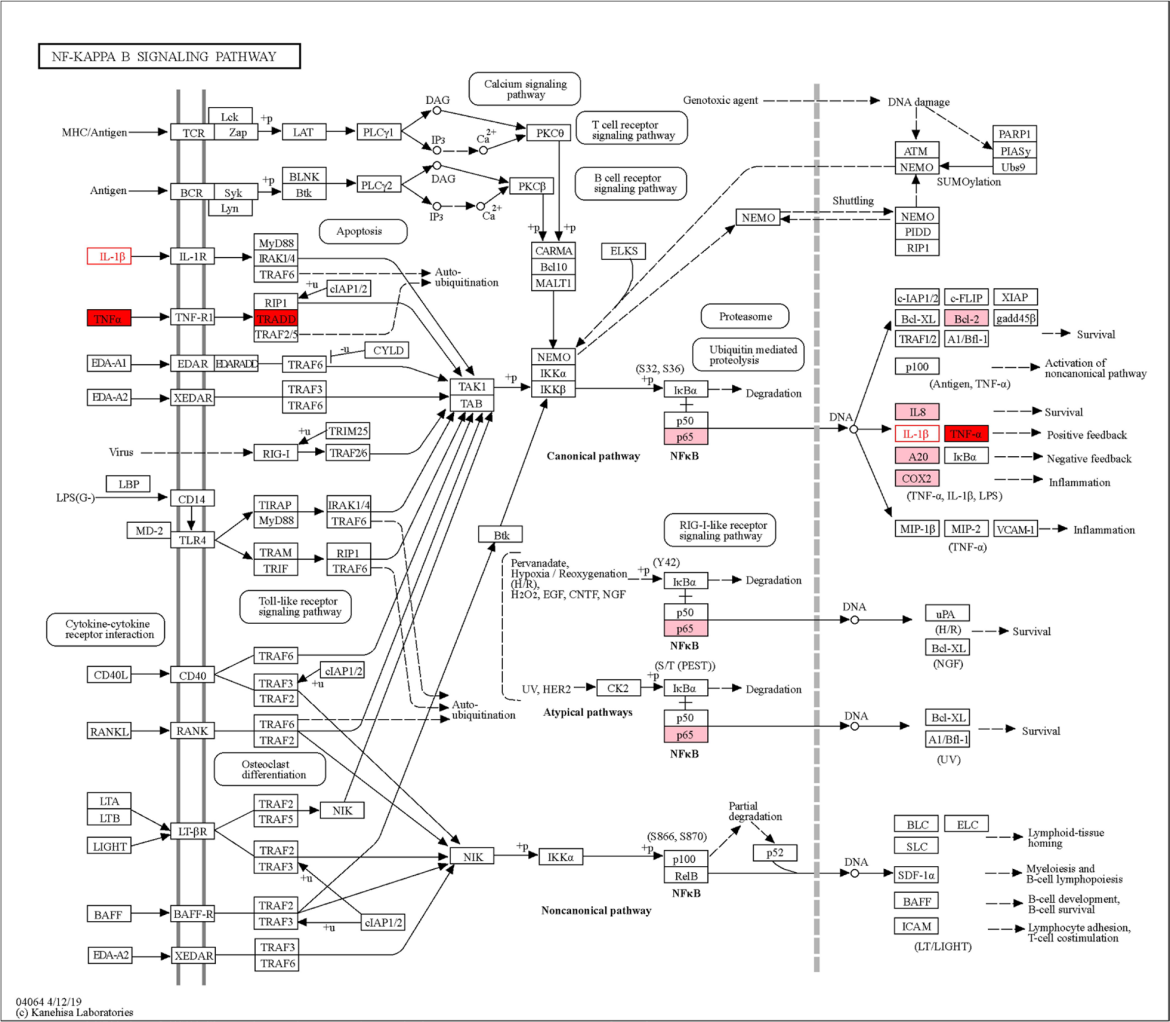
Schematic diagram of the NF-κB signaling pathway and its associated mechanisms. Factors such as IL-1β and TNF-α are used as promoters that mediate signal expression, leading to the expression of inflammatory proteins such as IL-18, IL-1β, COX-2, MMP and other inflammatory factors. and mediates the degradation of extracellular matrix and the death of NPCs

**Fig. 3:**
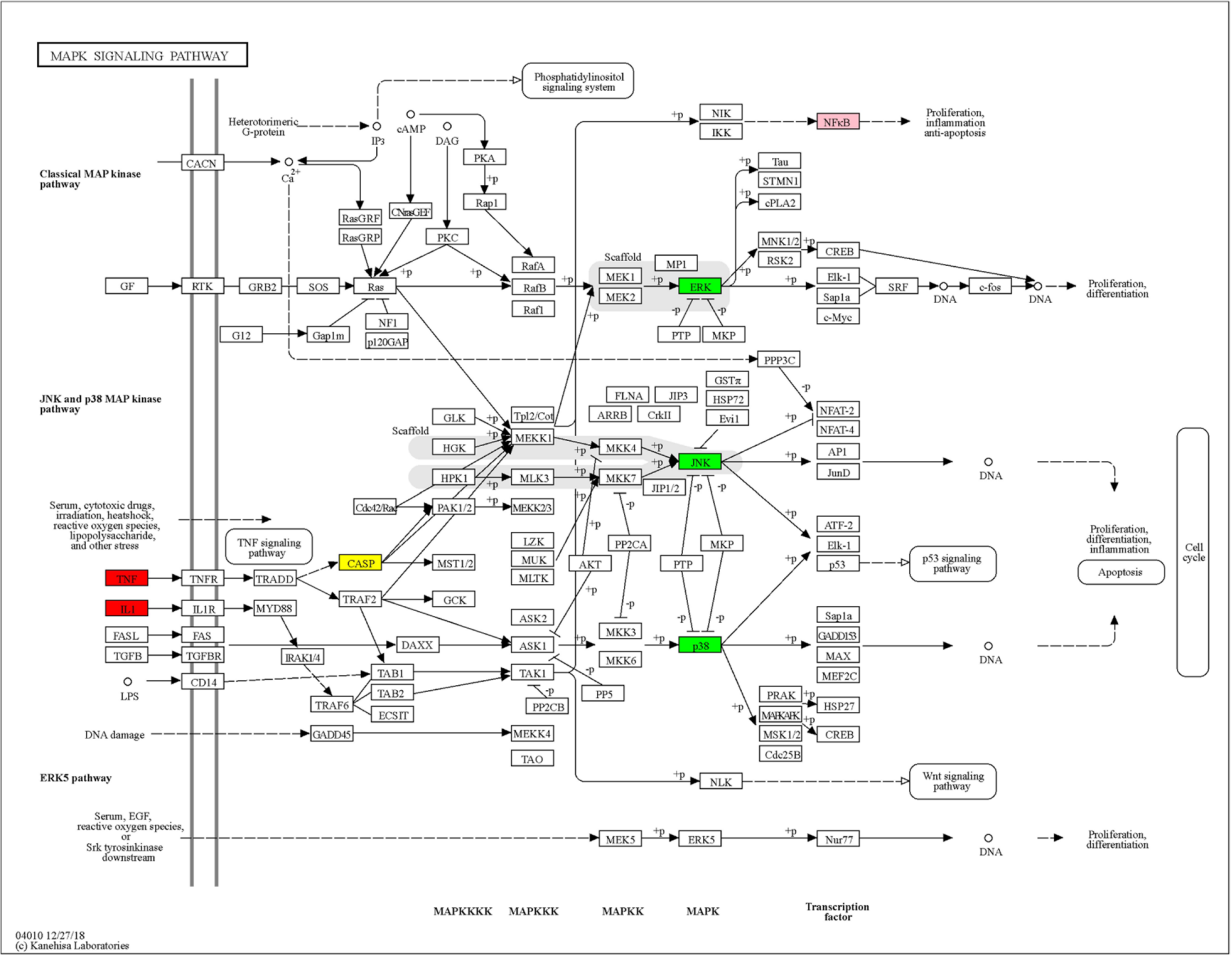
Schematic diagram of MAPK signaling pathway and its related mechanisms. Factors such as IL-1 and TNF are used as promoters that mediate signal expression, and JNK, p38 and ERK are three isoforms of the MAPK pathway. Ultimately, it mediates apoptosis of NPCs. This pathway exists in crosstalk with the NF-κB pathway and shares a common promoter

To verify that DHJSD is protective of NPCs by affecting the p38/MAPK signaling pathway, Liu used DHJSD alone to intervene in NPCs after IVDD modeling in an in vitro experiment, and the results showed that the levels of Bax and cleaved caspase-3 protein were reduced and the levels of Bcl-2 protein were significantly increased in NPCs treated with DHJSD. In addition, the apoptosis rate of NP cells in the DHJSD group was significantly reduced, and the NPCs treated with anisomycin, a p38/MAPK signaling pathway activator, after DHJSD intervention showed that anisomycin increased the level of p-p38 and reversed the differential expression of apoptotic factors in NP cells induced by DHJSD [16]. This suggests that the p38/MAPK pathway plays a key role in the protection of NPCs from apoptosis by DHJSD, which can block the excessive apoptosis of NPCs by inhibiting the activity of the p38/MAPK signaling pathway in NPCs. Stromal cell-derived factor-1 (SDF-1) is an important chemokine belonging to the C-X-C family, for which CXCR4 is the only receptor, and plays an important role in cell migration, proliferation, apoptosis and inflammatory response. The release of chemokines will promote the activity of inflammatory cytokines and thus increase the local inflammatory factor response. Our previous study showed that DHJSD could regulate the SDF-1/CXCR4 signaling axis to reduce NF-κB and IL-1β expression to regulate the apoptosis of NPCs, and the value-added ability of human NPCs treated with SDF-1 and different concentrations of DHJSD was determined by using CCK-8, and the results showed that the DHJSD mass concentration of 300 mg L^-1^ significantly increased the activity of NPCs treated with SDF-1 [30–33]. In summary, DHJSD can inhibit the apoptosis of NPCs by blocking the p38/MAPK signaling pathway, and can inhibit the apoptosis of NPCs by enhancing the activity of NPCs through the SDF-1/CXCR4/NF-κB signaling pathway, which can play a role in the therapeutic improvement of IVDD. In addition, after analyzing the KEGG database, we found that the MAPK signaling pathway is crosstalked with NF-κB signaling pathway during the activation of expression and shares a common promoter. The specific mechanisms involved are not fully understood and need to be further investigated (Figs. 2 and 3).

In addition to apoptosis, pyroptosis also plays an important role in IVDD. Pyroptosis is significantly different from apoptosis in both cytological changes and related mechanisms, pyroptosis is a form of inflammatory cell death that is triggered by various inflammasome, such as NOD-, LRR- and pyrin structural domain proteins (NLRP), and once these inflammasome are stimulated, downstream caspase1 (CASP1) will be cleaved, mediating the onset of pyroptosis [43]. Studies have shown that the expression levels of NLRP3 and its downstream targets caspase-1 and IL-1β are positively correlated with IVDD classification [44]. In the early stage of pyroptosis, small cationic permeation pores are formed in the plasma membrane, resulting in loss of ionic gradient, osmotic swelling, and cell lysis. The most striking feature of pyroptosis is that it is activated by CASP1 and is accompanied by the release of interleukin-1β and IL-18 inflammatory cytokines. There is a close association between inflammatory factors, especially IL-1β, and IVDD and a high expression in NPCs of IVDD patients.

To verify that NPCs undergo both apoptosis and pyroptosis during the IDD process, Bai isolated nucleus pulposus tissue and cells from patients with different grades of IDD (grade IV, grade V), and immunohistochemical staining showed the presence of CASP1 in NPCs, and flow cytometry analysis showed that NPCs from grade V patients had higher levels of reactive oxygen species (ROS) than those from grade IV patients. The expression of pyroptosis-related factors IL-1β, IL-18 and CASP1 was also higher in NPCs from grade V patients [45]. This suggests the presence of pyroptosis in the NPCs of IVDD patients, and the release of IL-1β and IL-18 inflammatory factors accompanying pyroptosis accelerates the progression of IVDD. Zhao demonstrated that activation of the ROS/NF-κB signaling pathway can induce NLRP3 inflammasome in NPCs and thus trigger pyroptosis. In in vitro experiments, human NPCs were exposed to 6 mM lactic acid solution, which regulates high levels of intercellular ROS via ASIC1 and ASIC3, and intracellular ROS levels were reduced by the ROS scavengers NAC (10 mM) and TEMPO (50 μm), and immunofluorescence and flow cytometry showed that the ROS scavengers reduced their scorch death levels. The increase in ROS activated the NF-κB signaling pathway, which promoted the expression of NLRP3. In addition, when intracellular ROS was absent or NF-κB signaling pathway was inhibited, NLRP3 activation and IL-1β release were reduced [31].

We previously found increased expression of pyroptosis proteins NLRP3 and GSDMD in degenerating NPCs and have shown that DHJSD regulates the SDF-1/CXCR4 signaling axis to suppress NF-κB pathway and IL-1β inflammatory factor expression, a classical inflammatory signaling pathway that can directly affect the intensity of inflammasome by activating the systemic inflammasome. It is also a regulatory protein of the cellular scorch NLRP3 pathway, mediating the NF-κB-NLRP3 pathway, which may also be a key pathway for DHJSD to regulate pyroptosis. We are currently conducting further experiments, and the exact mechanism and more relevant links between the signaling axes are yet to be further explored.

## DHJSD inhibits ECM degradation of NPCs

The NP has a significant role in the establishment and maintenance of the metabolism and structure of IVD tissue. Degeneration of the NPCs' ECM is one of the primary causes of IVDD. The mechanism by which its ECM degradation occurs is the stimulation of NPCs by various degenerative factors (e.g. compression and hypoxia), and the increased expression of inflammatory mediators is rapidly secreted into NP tissue and accelerated by the action of catabolic enzymes MMPs and adamts. Degradation of ECM downregulates collagen and proteoglycan expression, decreases ECM synthesis, and increases apoptosis of NPCs, which in turn leads to the development of IVDD [46, 47]. Therefore, inhibition of the degradation of ECM of NPCs is important for the prevention of IVDD.

Liu used Western blotting and real-time fluorescence quantitative PCR to detect the expression levels of ECM metabolic markers in NPCs [16]. Compared with the control group, the levels of type Collagen II, Sox-9, Aggrecan and mRNA were significantly lower in the NPCs of the IVDD model group, whereas the levels of ADAMTS-5, MMP-3, MMP-13 protein and mRNA were significantly higher. DHJSD treatment significantly increased the protein and mRNA levels of Sox-9, Collagen II and Aggregated glycans, whereas the protein and mRNA levels of ADAMTS-5, MMP-3 and MMP-13 were decreased [16]. Immunofluorescence staining results were consistent with the effect of DHJSD on Collagen II and MMP-3 expression [16]. The results showed that DHJSD inhibited the degradation of ECM of NPCs thereby providing protection to NPCs [48, 49].

**Fig. 4:**
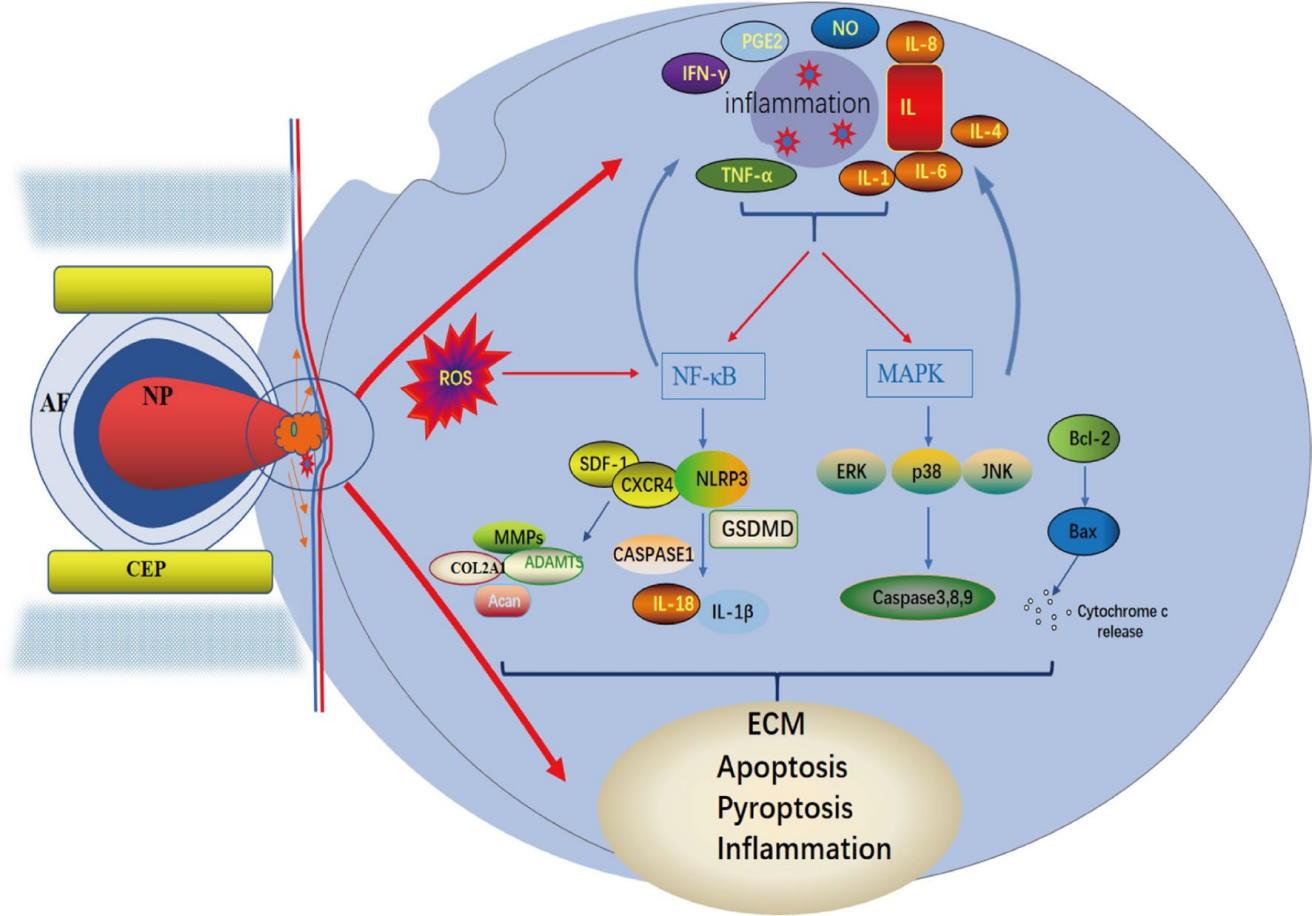
Schematic diagram of the cascade response to disc degeneration. In degenerated discs, NPCs are stimulated by various degenerative factors that induce the release of inflammatory mediators (IL, INF-γ, TNF-α, etc.), and inflammatory cytokines activate sensory neurons and produce pain. IL-1β and TNF-α act as promoters to activate NF-κB and MAPK pathways and are involved in the production of cytokines, chemokines and extracellular matrix degradation factors in IVD. leading to inflammation and extracellular matrix degradation as well as death of NPCs

We performed in vitro culture by isolating disc tissue from patients with primary IVDD and normal disc tissue from patients with lumbar spine fractures. The results showed that the application of DHJSD significantly antagonized the production of sdf-1-induced pro-inflammatory cytokines in NPCs and increased the accumulation of Agg and Collagen II formation [16]. Activation of the NF-κB signaling pathway is closely related to ECM degradation, and upon activation of this pathway, a variety of inflammatory cytokines are released, such as TNF-α, IL-2, IL-6, IFN-γ and catabolic enzymes, including MMP-3, MMP-9, MMP-13, ADAMTS-4 and ADAMS-5. Meanwhile, the expression of type II collagen α1 (COL2A1) and aggregates (Acan) was reduced, which led to ECM degradation [16, 50]. DHJSD significantly reduced the sdf-1-induced increase in CXCR4 and later intervened in sdf-1-induced NPCs using a combination of DHJSD, CXCR4-siRNA and NF-κB inhibitor (BAY11-7082) with the same effect, suggesting that DHJSD inhibits ECM degradation by synergistically targeting multiple molecules in the SDF-1/CXCR4/NF-κB pathway [6, 51] (Fig. 4).

## DHJSD improves intestinal flora and serum metabolites to treat IVDD

**Fig. 5:**
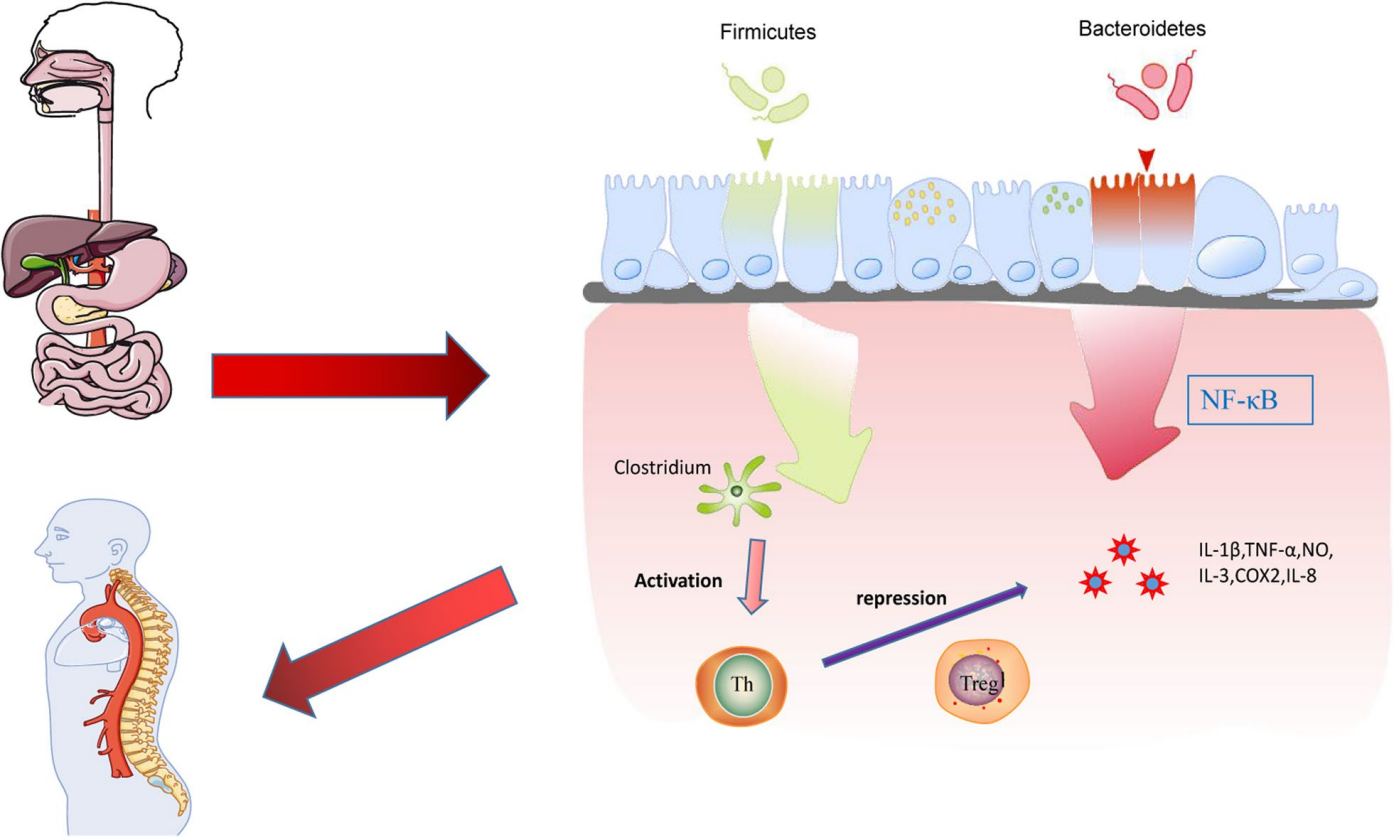
Diagram of the relationship between intestinal flora dysbiosis and intervertebral disc degeneration. The Bacteroidetes acts on the NF-κB signaling pathway to produce an inflammatory response, and Clostridium perfringens in the Firmicutes, which slows disc degeneration by modulating immunity with the release of anti-inflammatory factors

Dysbiosis of intestinal flora and altered serum metabolic phenotype may be closely related to disc degeneration, and intestinal flora and serum metabolism share a common metabolic pathway of change [52, 53]. IVDD can cause dysbiosis of intestinal flora, alteration of serum metabolic phenotype and related metabolic pathways, while DHJSD can regulate intestinal flora, serum metabolites and metabolic pathways and inhibit disc degeneration [52]. Numerous studies have shown that changes in intestinal flora are closely associated with orthopedic metabolic diseases [54, 55]. Bone disease can be brought on by gut flora dysbiosis, which can disrupt dynamic bone homeostasis and bone metabolism. The functioning mechanism may involve the gut flora, intestinal permeability, metabolites, and the regulation of immune response, hormones, and neurotransmitters [57–60]. We found that Bacteroidetes, Firmicutes and Proteobacteria were the main groups causing IVDD by analyzing the differences in intestinal flora species. Among them, the Bacteroidetes belongs to conditional pathogenic bacteria, and the Firmicutes belongs to human probiotics. Studies have shown that the Bacteroidetes can act on NF- κB signaling pathways to produce inflammatory responses. And the Firmicutes can promote polysaccharide fermentation [61], involved in maintaining the balance and stability of the intestinal flora.An increase in the relative abundance of the Bacteroides and a decrease in the relative abundance of the Firmicutes are associated with intestinal inflammation [62]. Among the resident flora, Clostridium perfringens belongs to the Firmicutes, which is associated with Th cell development and Treg cell induction, suggesting that intestinal flora may regulate the immune system and improve disc degeneration through the release of molecules with immunomodulatory and anti-inflammatory functions [63]. We showed by macrogenomic analysis that DHJSD can increase the abundance of rat intestinal flora, improve the composition of the flora, upregulate the Firmicutes and the Proteobacteria, downregulate the Bacteroidetes to play a biological role, thus inhibiting intervertebral disc degeneration (Fig. 5).

**Fig. 6:**
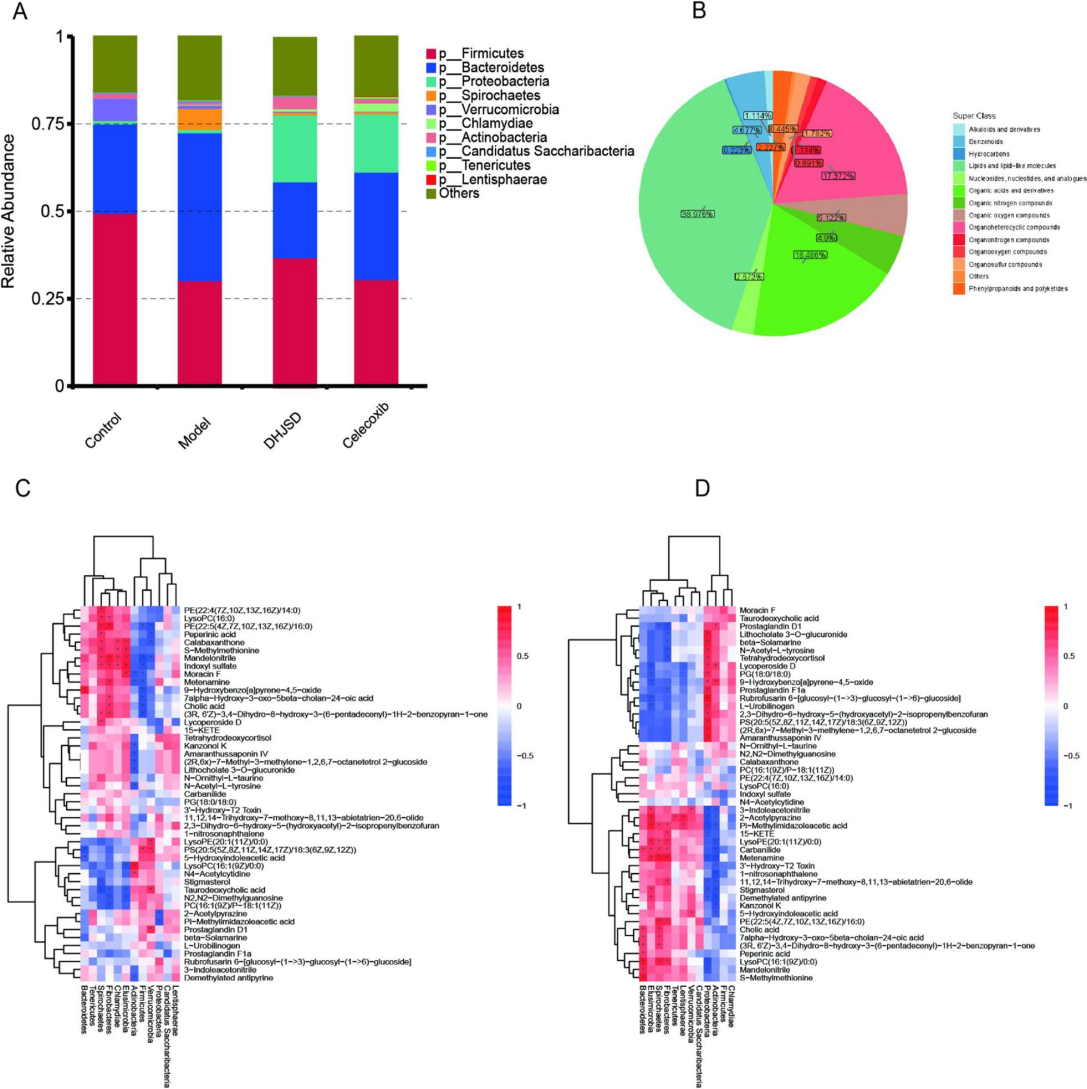
**A** Changes in abundance of different intestinal flora based on gate-level IVDD and DHJSD treatment. **B** Proportion of serum metabolites classified in IVDD based on serum metabolite analysis. **C** Heat map of association analysis between intestinal flora and serum metabolites in normal group and IVDD model group. **D** Heat map of association analysis between intestinal flora and serum metabolites in IVDD model group and DHJSD treatment group (red indicates positive correlation, blue indicates negative correlation *P < 0.05)

Through macro-genetic analysis of intestinal flora, our group found that DHJSD may regulate TNF, NF-kappa B, PI3K-Akt, MAPK signaling pathways through key proteins such as CASP8, TNF-α and IL-3 to slow down the apoptosis of NPCs and thus treat intervertebral disc degeneration [6, 48]. Serum metabolic analysis showed that DHJSD could improve the degenerative process of intervertebral discs through metabolic pathways such as glycine, serine and threonine metabolism, citric acid cycle, arginine biosynthesis, lysine biosynthesis, methane metabolism, valine, leucine and isoleucine biosynthesis, and thioglucoside biosynthesis. During amino acid metabolism, proteoglycan is a hydrophilic linker protein and core protein containing high amounts of acidic amino acids that make up cartilage and maintain the anatomy and function of the intervertebral disc [64]. Non-targeted metabolomics based on serum metabolites show that DHJSD mainly improves disc degeneration in rats by regulating amino acid metabolism and carbohydrate metabolism. Glutamate and alanine in the amino acid metabolic pathway, the pathways of carbohydrate metabolism sucrose and glucose 1-phosphate may play a major role. Amaranth saponin IV, stigmasterol, and lysophospholipid (LysoPC(14:0/0:0)) metabolites may also play important regulatory roles (Fig. 6B). Metabolic pathway analysis revealed that glutamate metabolism, glycerophospholipid metabolism, citric acid cycle, glycine, serine and threonine metabolism, valine, leucine and isoleucine biosynthesis and degradation, niacin and nicotinamide metabolism, sphingolipid metabolism, aminoacyl-tRNA biosynthesis, and tryptophan metabolism are closely associated with DHJSD for disc degeneration. Both intestinal flora and serum metabolite analysis showed that DHJSD treatment of disc degeneration was associated with glycine and serine as well as threonine metabolism, citric acid cycle, valine, leucine and isoleucine biosynthesis, suggesting that this may be a common metabolic pathway that interacts between intestinal flora and serum metabolites. In addition, we found a close association between intestinal flora and serum metabolites [65]. In the treatment of IVDD, DHJSD can positively modulate intestinal flora and serum metabolites or negatively modulate intestinal flora and serum metabolites [61, 66] (Fig. 6C, D). In summary, DHJSD regulates intestinal flora and serum metabolites through multiple targets, metabolic differences, and metabolic pathways in the treatment of intervertebral disc degeneration [65]. However, the specific mechanisms and metabolic pathways of these differentially expressed substances in IVDD, as well as the mechanisms of their regulation by DHJSD, need to be further investigated.

## Discussion

IVDD has become a serious problem endangering public health and posing a heavy burden to the society and medical system. As a hot research area of orthopedic diseases, its pathogenesis have not been fully elucidated, and there is still a lack of treatment modalities to solve the problem at its root. Today's clinical medical research has demonstrated that DHJSD has significant clinical effects in the treatment of IVDD and has few adverse effects. Its specific mechanism involves inhibiting inflammatory response and reducing NPCs death, inhibiting ECM degradation, and improving intestinal flora.

DHJSD can inhibit the production of pro-inflammatory mediators not only through the SDF-1 /CXCR4/NF-κB pathway, but also through the MAPK pathway to inhibit the release of inflammatory factors IL-1β and TNF-α. In reducing the death of NPCs and inhibiting ECM degradation, DHJSD could inhibit the activity of p38/MAPK signaling pathway, regulate the SDF-1/CXCR4 signaling axis to reduce the expression of NF-κB and IL-1β, MMP-3, MMP-13, etc., and increase the expression of type II Collagen, Sox-9, Agg, etc., to block the excessive apoptosis of NPCs and inhibit ECM degradation, in addition, DHJSD can mediate NF-κB-NLRP3 pathway to prevent pyroptosis. Moreover, through macro-genetic and metabolomic analysis, we obtained that DHJSD can positively or negatively regulate intestinal flora and serum metabolites through multi-target, multi-metabolic differences and multi-metabolic pathways, enhance the abundance of beneficial bacteria gate to regulate the immune system and release anti-inflammatory factors for the treatment of IVDD. In summary, DHJSD can prevent and control the occurrence and development of IVDD by modulating inflammatory factors, inhibiting apoptosis, pyroptosis, and ECM degradation of NPCs, and improving intestinal flora and serum metabolites in various ways. These mechanisms of action involve multiple signaling pathways, and the activation of MAPK signaling pathway plays an important role and there is crosstalk with NF-κB signaling pathway, but the specific mechanism of action needs to be further explored. In addition, DHJSD plays an important role in the prevention and treatment of IVDD with altered oxidative stress within the NP, but the mechanism is not clear. A series of questions on whether there are more ways of death within the NPCs in degenerated discs and whether DHJSD can regulate these cell death ways remain to be confirmed by further studies. Even though there has been a great deal of research, the components and targets of action of the herbal compound DHJSD are complex and not entirely known. Second, it is unclear which DHJSD active components can get to the intervertebral discs and function as an organ devoid of blood vessels. The mechanism study of DHJSD for IVDD will provide a theoretical basis and scientific basis for the use of TCM. Our team will further deepen the exploration of the connection of related mechanisms in this field.

## Data Availability

The datasets used and analyzed during the current study are available from the corresponding author upon reasonable request.
